# Immunisation status of children and adolescents with a new diagnosis of inflammatory bowel disease

**DOI:** 10.1186/s12879-021-06976-x

**Published:** 2022-01-04

**Authors:** Timothy Ford, Margie Danchin, Alissa McMinn, Kirsten Perrett, George Alex, Nigel W. Crawford

**Affiliations:** 1 Perth Children’s Hospital, Nedlands, WA Australia; 2 Murdoch Children’s Research Institute (MCRI), Parkville, Melbourne, Australia; 3 Immunisation services & General Medicine, RCH, Melbourne, Australia; 4 Department Paediatrics, University of Melbourne, Parkville, Australia; 5 Gastroenterology Department, RCH, Melbourne, Australia

## Abstract

**Background:**

Patients with Inflammatory Bowel Disease (IBD) are at increased risk of serious infections, including vaccine preventable diseases. Current evidence suggests uptake of additional recommended special risk vaccinations is low. Identification of IBD patients prior to commencing immunosuppressive therapy allows for optimisation of vaccination, including timely administration of live-attenuated and additional recommended vaccines, such as influenza and pneumococcal vaccines.

**Methods:**

Paediatric patients (0–18 years) seen at the tertiary Royal Children’s Hospital, Melbourne, Australia, with a recent diagnosis of IBD were referred by the Gastroenterology Unit to our Specialist Immunisation Clinic (SIC) for assessment and provision of routine and special risk vaccines. Data was collected via a standardised REDCap questionnaire completed in or post attendance at the SIC and included serology results where available.

**Results:**

Sixty-nine paediatric patients were recruited to the study between 2014 and 2017. Median age at IBD diagnosis was 11.25 years (IQR 4.64 years), with median time between diagnosis and SIC review of 0.88 years (IQR 2.84 years). At initial review 84.1% (58/69) of patients were up to date with vaccines on the Australian National Immunisation Program (NIP) schedule. Of those who were tested, serological evidence of immunity was demonstrated in 38.3% (23/60) of patients for Hepatitis B, 66.7% (36/54) for measles, 51.9% (28/54) for rubella and 41.9% (26/62) for Varicella Zoster Virus. Prior to SIC review 47.8% (33/69) had additional vaccinations and 92.8% (64/69) had vaccinations administered in the 12 months following SIC assessment. The Pneumococcal conjugate vaccine (76.8%, 53/69) was the most commonly administered vaccine after SIC review, followed by influenza vaccine (69.6%, 48/69). Within 12 months of SIC review 43.5% (30/69) of patients had completed the schedule and were up-to-date as recommended by the SIC.

**Conclusions:**

Children with IBD and other special risk groups can benefit from early referral to a SIC team to ensure optimal administration of routine and additionally recommended vaccines, especially live and additional special risk vaccines. The value of optimising immunisations could also be applied to other special risk groups, including adult IBD cohorts, particularly those commencing newer biologic immunosuppressive medications.

## Background

The incidence of paediatric Inflammatory Bowel Disease (IBD) has risen internationally and has been well described in the Australian context [1, 2]. Retrospective analyses from Victoria, Australia, have shown a marked increase in incidence of both Ulcerative Colitis (11-fold from 1990 to 2010) and Crohn’s Disease (15-fold from 1970 to 2000) [1, 2] Patients with IBD are at increased risk of serious infections, including vaccine preventable diseases (VPD) [3]. This increased infection risk stems from a number of factors including the immunomodulatory effects of the disease process, immunosuppressive therapy, suboptimal nutrition and nosocomial infections associated with surgery, parenteral nutrition and hospital attendances [4]. Immunosuppressive agents such as biologics are increasingly being used early in the disease course and have been associated with severe infections [5]. This risk appears increased when combining two or more agents [6]. There have been case reports of serious VPD in immunosuppressed IBD patients, including severe varicella [7, 8], influenza [9] and pneumococcal disease [10]. High rates of cervical dysplasia have been described in adult female patients with IBD, highlighting the importance of timely Human Papillomavirus (HPV) vaccination [11, 12].

Current recommendations outline that children and adolescents with IBD should receive additional doses of pneumococcal conjugate vaccine (PCV) and a yearly seasonal influenza vaccine [13]. A 2007 retrospective audit of 101 patients identified using a state-based IBD register in Victoria, Australia, showed that 90% were up to date with vaccinations according to the routine schedule [14]. However, uptake of additional recommended vaccines in this group was low, with only 10% having ever had an influenza vaccination, and 5% an additional pneumococcal vaccination [14]. A recent European case-control study showed that influenza immunisation rates were lower in children with IBD than in the general paediatric population, with fear of vaccine side effects identified as the most significant reason for this difference [15].

Administration of live-attenuated vaccines, such as varicella and measles-mumps-rubella (MMR), is usually considered contraindicated in immunosuppressed patients. Identification of IBD patients prior to immunosuppression allows for optimisation of vaccination, including timely administration of live vaccines at the same time as additional recommended vaccines. This process also assists to minimise the problem of diminished immune response due to immunomodulatory therapies. This is particularly important for patients on a combination of tumour necrosis factor (TNF) inhibitors and immunomodulators, which have been shown to result in impaired immune responses to both PCV and pneumococcal polysaccharide vaccines (PPV) [16, 17].

In this study we aimed to determine the immunisation status of paediatric patients newly diagnosed with IBD at the time of review at the SIC, according to the Australian National Immunisation Program (NIP) schedule. Secondary objectives included timeliness of administration of NIP and special risk vaccines, serum immune response as determined by serological testing for Hepatitis B Virus (HBV) and live-attenuated vaccines [Varicella Zoster Virus (VZV) and (MMR)], use of immunosuppressive agents, uptake of additional recommended vaccines, and any adverse events following immunisation (AEFI). We hypothesized that immunisation uptake would be suboptimal and that this information would allow for identification of commonly missed vaccines and provide an opportunity for optimisation of immunisation prior to commencement of immunosuppressive therapy.

### Methods

The study was conducted at the Royal Children’s Hospital (RCH), Melbourne, Australia—a large tertiary paediatric centre, with a specialist gastroenterology service. Data was collected both retrospectively and prospectively between January 2014 and July 2017. Participants recruited were paediatric patients (0–18 years) recently diagnosed with IBD, who were referred from the Gastroenterology Clinic to the SIC, which was the standard practice in the hospital. Retrospective cases were identified by reviewing the RCH IBD patient register and cross-checking this with the SIC database. At SIC review a standardised questionnaire to collect data pertaining to demographic details, disease type and co-morbidities, IBD treatment, immunisation status, and risk factors for tuberculosis was utilised. Immunisation status was determined by history and reviewing the Australian Children’s Immunisation Register (ACIR), Australian Immunisation Register (AIR, which replaced the ACIR on 30 September 2016), RCH Immunisation Program Service (ImPS) database and the RCH electronic medical record (since 30 April 2016). Serological testing was then requested, including for MMR, VZV and HBV. Correlates of protection were determined as hepatitis B surface antibody > 10 IU/ml for HBV and rubella IgG antibody > 10 IU/ml for rubella. Measles, mumps and VZV serology were reported as either positive (immune), equivocal, or negative (non-immune). Additional vaccinations were offered according to serological results, the Australian NIP schedule and Australian Immunisation Handbook (AIH) guideline on special risk groups [13]. Review of adverse events following immunisation (AEFI) was conducted 1 year after initial SIC review by interrogating the Surveillance of Adverse Events Following Vaccination in the Community (SAEFVIC) database—the spontaneous (passive) surveillance system for all notified AEFI in the RCH jurisdiction of Victoria.

Data were entered into a REDCap (Research Electronic Data Capture) database hosted at the Murdoch Children’s Research Institute (MCRI) and results were analysed using SPSS (IBM SPSS Statistics for Windows, Version 25). Proportions of up-to-date status and additional vaccines administered were compared using a Pearson chi-square test with a P value < 0.05 considered statistically significant. Ethics approval was obtained from the Royal Children’s Hospital Human Research Ethics Committee HREC#35060A.

## Results

Sixty-nine patients diagnosed with IBD were reviewed in the SIC between January 2014 and July 2017. Demographic details are summarised in Table 1. The majority of patients were male (60.9%), 58% had Crohn’s Disease and 34.8% Ulcerative Colitis. The median age at diagnosis of IBD was 11.25 years (IQR 4.64) and the median age at first SIC review was 13.12 years (IQR 4.61 years). 68.1% patients resided in a major city according to the Australian Statistical Geography Standard (ASGS) system [18].

**Table 1:** Patient characteristics

	n (%)
Gender, female	27 (39.1%)
Diagnosis	
Ulcerative colitis	24 (34.8%)
Crohn’s disease	40 (58%)
Indeterminate	5 (7.2%)
Age at diagnosis, years, median (IQR)	11.25 (4.64)
Age at immunisation service review, years, median (IQR)	13.12 (4.61)
Time between diagnosis and SIC review, years, median (IQR)	0.88 (2.84)
Country of birth Australia	62 (89.9%)
Rural status^a^	
Major city	47 (68.1%)
Inner regional	19 (27.5%)
Outer regional	2 (2.9%)
Remote	0 (0%)
Very remote	1 (1.4%)

^a^Determined utilising the five point ASGS system [18]

## Baseline immunisation status

At initial SIC review, 84.1% patients were up to date with vaccinations according to the NIP schedule for age (see Table 2). Meningococcal C vaccine had been received by 68.1% and 59.1% (26/44) of eligible patients received an HPV vaccine, in keeping with the age range of the cohort, and noting that introduction into the routine Victorian schedule only occurred in 2003 for Meningococcal C vaccine, 2007 for HPV vaccine for females, and 2013 for HPV vaccine for males [19].

**Table 2:** Baseline immunisation status in children and adolescents with IBD (N = 69)

		n (%)	
Immunisations up to date with routine Australian NIP schedule		58 (84.1%)	
Past history of varicella infection		10 (14.5%)	
Previous vaccination history			
Previous HPV vaccine given (eligible ≥ 12)		26/44 (59.1%)	
Previous Meningococcal C vaccine given^a^		47 (68.1%)	
Previous Influenza vaccine given		23 (33.3%)	
Serologically confirmed immune status (number tested)			
At least 1 serological test		64/69 (92.8%)	
All 5 serological tests		52/69 (75.4%)	
HBV immune (60)		23/60 (38.3%)	
Measles immune (54)		36/54 (66.7%) Equivocal 2/54 (3.7%)	
Mumps immune (54)		39/54 (72.2%) Equivocal 3/54 (5.6%)	
Rubella immune (54)		28/54 (51.9%) Equivocal 7/54 (13.0%)	
VZV immune (62)		26/62 (41.9%) Equivocal 6/62 (9.7%)	
Tuberculosis status			
BCG vaccine		6 (8.7%)	
Travel to TB high-incident country		17 (24.6%)	
TB screening at or before SIC		39 (56.5%)	
QuantiFERON (n = 38)		Negative: 33/38 (86.8%) Indeterminate: 5/38 (13.2%) Positive: 0	
Tuberculin skin test (n = 1)		Negative: 1	
QuantiFERON requested but not done		N = 2	
Patients with travel to high incident country (n = 17)		15 (88.2%)	p = 0.155
Patients on biologic agent (n = 16)		14 (87.5%)	p = 0.009

*HPV* Human Papillomavirus, *HBV* Hepatitis B Virus, *VZV* Varicella Zoster Virus

^a^The meningococcal C vaccination was introduced as routine to the schedule in Victoria in January 2003. A catch-up program was offered to 1–19 year olds until 2006 [19]

## SIC review and vaccine administration timeliness

The median time between diagnosis and SIC review was 10.5 months (IQR 2.8 years). During this period, 47.8% patients had at least one vaccination, with the most commonly received vaccines being influenza vaccine (51.5%; 17/33), diphtheria-tetanus-acellular pertussis (dTap; 39.4%, 13/33), HPV vaccine (36.4%; 12/33), and VZV vaccine (33.3%; 11/33). Only 18.1% (6/33) patients received an additional pneumococcal vaccine (either conjugate or polysaccharide), prior to SIC review.

## Serological status of vaccine preventable diseases

92.8% patients had at least one serological test recorded at initial SIC review (see Table 2). Serological testing identified 38.3% (23/60) to be HBV immune, 66.7% (36/54) measles immune, 72.2% (39/54) mumps immune, 51.9% (28/54) rubella immune and 41.9% (26/62) varicella immune. Serologically confirmed rubella immunity was more common in females (16/28, 57.1%). Of the ten children with a reported history of previous varicella infection, only one was found to be non-immune on serological testing.

39/69 (56.5%) patients were screened for tuberculosis following SIC review, including 15/17 (88.2%) who had travelled to a tuberculosis high-incident country and 14/16 (87.5%) who were on a biologic agent (see Table 2). 38/39 (97%) of these patients had an interferon-gamma release assay (QuantiFERON-TB Gold assay), of which there were 5/38 (13.2%) indeterminate results and no positive results.

## Anti-inflammatory and immunosuppressive medications

At the time of SIC review, 42% of patients had used an aminosalicylate. Overall, 78.3% had used one or more of a: corticosteroid (29/69; 42%), immunomodulator, including one of either azathioprine, 6-mercaptopurine or methotrexate (41/69; 59.4%), or, an anti-TNF agent (16/69; 23.2%) (see Table 3).

**Table 3:** IBD anti-inflammatory andimmunosuppressive treatment

	n (%)
None recorded	7 (10.1%)
Aminosalicylates	29 (42%)
Mesalazine	24 (34.8%)
Salazoprine	5 (7.2%)
Steroids	44 (63.8%)
Methylprednisolone	16 (23.2%)
Prednisolone	42 (60.9%)
Budesonide	2 (2.9%)
Immunosuppressor	41 (59.4%)
Azathioprine	27 (39.1%)
6-Mercaptopurine	2 (2.9%)
Methotrexate	12 (17.4%)
On-going immunosupressor at time of SIC review	37/41 (90%)
Biologic agent (infliximab)	16 (23.2%)
On-going biologic agent at time of SIC review	14/16 (87.5%)
Highest level of immunosuppression ever	
None/NSAID	14 (20.3%)
Steroidimmunosuppressor	38 (55.1%)
Biologic agent	16 (23.2%)

*NSAID* non-steroidal anti-inflammatory drug

## Uptake of additional recommended vaccines

Following SIC review, 98.6% patients were offered 356 vaccines (median 5, IQR 2). The most commonly recommended vaccines were PCV (60/69, 87%), PPV (57/69, 82.6%) and an influenza vaccine (51/69, 73.9%). Sixty-four (92.8%) patients were administered 307 vaccines (median 5, IQR 3) in the 12 months following SIC review. The most commonly received vaccinations during this period were PCV (53/69, 76.8%), PPV (38/69, 55.1%), an influenza vaccine (48/69, 69.6%) and a Hepatitis A Virus (HAV) vaccine (36/69, 52.2%). 85.5% (63/69) of patients received at least one pneumococcal vaccine following SIC review. Only 43.5% of patients had completed the SIC recommended vaccination schedule at 12 months following SIC review. No statistically significant relationship was identified between immunisation status and vaccination recommendation or uptake following SIC review (see Table 4).

**Table 4:** Additional vaccinations received by IBD cohort (N = 69)

	Received after IBD diagnosis and before SIC review n (%)	Received within 12 months of SIC review n (%)	Received any time since IBD diagnosis n (%)
Any vaccination	33 (47.8%)	64 (92.8%)	67 (97.1%)
Any pneumococcal vaccination	6 (8.7%)	59 (85.5%)	63 (91.3%)
PCV	6 (8.7%)	53 (76.8%)	59 (85.5%)
PPV	1 (1.4%)	38 (55.1%)	39 (56.5%)
Influenza	17 (24.6%)	48 (69.6%)	55 (79.7%)^a^
VZV	11 (15.9%)	18 (26.1%)	29 (42%)
MMR	6 (8.7%)	16 (23.3%)	22 (31.9%)
HPV	12 (17.4%)	19 (27.5%)	25 (36.2%)^a^
dTap	13 (18.8%)	17 (24.6%)	30 (43.5%)
HBV	3 (4.3%)	27 (39.1%)	29 (42%)^a^
HAV	6 (8.7%)	36 (52.2%)	37 (53.6%)^a^
Meningococcal ACWY	1 (1.4%)	15 (21.7%)	16 (23.2%)
Meningococcal B	0	19 (27.5%)	19 (27.5%)
Other	7^b^ (10.1%)	3^c^ (4.3%)	10 (14.5%)
Total vaccinations (median; IQR)	84 (0; 0–2)	307 (5, 3–6)	391 (5; 4–7)
Completed SIC recommended schedule within 12 months			30 (43.5%)

*PCV* pneumococcal conjugate vaccine, *PPV* pneumococcal polysaccharide vaccine, *VZV* Varicella Zoster Virus, *MMR* measles, mumps, rubella, *HPV* Human Papillomavirus, *dTap* diphtheria, tetanus, acellular pertussis, *HBV* Hepatitis B Virus, *HAV* Hepatitis A Virus, *Infanrix-IPV* diphtheria, tetanus, acellular pertussis, inactivated poliovirus vaccine, *Infanrix-Hexa* diphtheria, tetanus, acellular pertussis, Hepatitis B Virus, poliovirus and *Haemophilus influenzae* type B vaccine

^a^Some patients will have received multiple of these vaccines in the period between IBD diagnosis and analysis at 12 months following SIC review

^b^Other: typhoid (1), Infanrix-IPV (6)

^c^Other: typhoid (1), Infanrix-IPV (1), Infanrix-Hexa (1)

There were forty-three patients who had either negative, equivocal or unknown VZV serology. Of these, 55.8% (24/43) received VZV vaccination following IBD diagnosis. Utilising serology results, 44.2% (19/43) were recommended VZV vaccination at SIC review and 89.5% (17/19) actually received the vaccine following this clinic review. There were forty-five patients who had non-immune, equivocal or unknown serological results for one or more of either measles, mumps or rubella. Of these, 40% (18/45) received an MMR vaccine following IBD diagnosis, with 35.5% (16/45) receiving the vaccine after SIC review. Of the forty-six (66.7%) patients who were non-immune to HBV, 60.1% (28/46) received HBV vaccine following IBD diagnosis, with 63% (29/46) recommended HBV vaccination at SIC review and 54.2% (26/46) receiving the vaccine after SIC review. Patients who had demonstrated absence of or equivocal immunity on serological testing were more likely to receive vaccination than those who had unknown serology for MMR (p = 0.012) and HBV (p = 0.018), but not VZV (p = 0.953) (see Table 5).

**Table 5:** Impact of serology results on vaccination uptake in IBD cohort

Vaccine administered	Serology result n/d^a^ (%)			p value
	Non-immune or equivocal	Unknown	Total	
VZV	20/36 (55.5%)	4/7 (57.1%)	24/43 (55.8%)	p = 0.953
MMR	16/30 (53.3%)	2/15 (13.3%)	18/45 (40%)	p = 0.012
Measles	12/18 (33.3%)	2/15 (13.3%)	14/33 (42.4%)	p = 0.004
Mumps	12/15 (80%)	2/15 (13.3%)	14/30 (46.7%)	p = 0.001
Rubella	15/26 (57.7%)	2/13 (13.3%)	17/39 (43.6%)	p = 0.008
HBV	26/37 (70.3%)	2/9 (22.2%)	28/46 (60.1%)	p = 0.018

^a^Number of patients with relevant serology result who were vaccinated/number of patients with relevant serology result

Additional vaccines were recommended to the household contacts of 69.6% of patients, including influenza (44/69, 63.8%), dTap (27/69, 39.1%) and varicella (10/69, 14.5%).

## Post-SIC review

Two significant AEFI were reported to SAEFVIC during the prescribed study follow-up period. A 16-year-old female developed a severe injection site reaction (ISR) within 2 h of 13 valent PCV (Prevenar13®) administration. Supportive therapy was recommended and hospital admission was not required. A 5-year-old female developed a severe ISR and fever within eight hours of 23 valent PPV (Pneumovax®) and influenza vaccination (Fluarix®). She was assessed by her General Practitioner and provided supportive treatment, with no hospitalisation required.

## Discussion

This is the first Australian study that assessed referral and assessment of newly diagnosed paediatric IBD patients within a specialised immunisation clinic. Immunisation rates according to the standard Australian NIP schedule at the time of SIC review in this cohort were slightly lower (at 84.1%) than the Victorian baseline rates, which were 92.5–94.6% at 5 years of age over the years of the study [20]. Timeliness of SIC review in relation to diagnosis of IBD was variable, with the median time between diagnosis and review being just above 10 months. Additionally, there was variability in the uptake of vaccinations during the time between diagnosis and SIC review, with only 33 patients (47.8%) receiving an additional vaccination during this time. Concerningly, at the time of SIC review, only 8.7% of patients had had an additional pneumococcal vaccine, which is recommended by the AIH special risk group guideline [13].

These issues are consistent with previously published literature on immunisation status assessment in the initial period following IBD diagnosis [21, 22]. One review of American Gastroenterologists demonstrated that only 63% assessed immunisation status at the time of IBD diagnosis, with assessment limited primarily to influenza (78%), hepatitis B (84%), and varicella (82%), and fewer than 55.5% reviewing the status of other immunisations [21].

The utilisation of serological assessment of immunisation status was common in this study, with 92.8% of patients having at least one serological test and 75.4% having all of HBV, MMR and VZV serological titres recorded. These investigations demonstrated relatively low rates of serologically confirmed immunity for HBV, MMR and VZV despite evidence of previous primary immunisation. As our data did not specify the timing of serological assessment, it is unclear if this is a result of inadequate serum immune response immediately following vaccination, or if it represents waning of antibodies and immune protection over time. It may also relate to the limitations of laboratory antibody testing for live-attenuated vaccines, but despite these limitations, it did appear that serological results were important in guiding vaccination recommendations and correlated with subsequent vaccine uptake. Patients demonstrating absence of immunity on serological tests for HBV, measles, mumps and rubella were statistically more likely to receive the relevant vaccine than those who had unknown serological results in our cohort.

Vaccine efficacy remains an area of uncertainty in the IBD cohort [23]. Reduced serum immune response to vaccination is likely due to a combination of immunological alterations generated by the disease itself and to the immunomodulatory treatments that most IBD patients invariably require [24, 25]. Some vaccines fare better than others, for example the serum immune response to HPV vaccine seems similar to that observed in healthy populations [24]. Whilst studies assessing the response to VZV and MMR are scarce, reduced immunogenic response to other important vaccines, including, HBV, influenza and pneumococcal vaccines has been reported [16, 17, 23, 24]. In a recent meta-analysis of studies that reviewed HBV vaccination in patients with IBD, only 61% demonstrated an immunogenic response to HBV vaccination [23]. A positive response to HBV vaccination was more likely in those with a younger age and those who were vaccinated during remission, with no difference seen when different immunotherapies were compared [23]. De Bruyn et al. [26] demonstrated that serologic protection to influenza vaccine is achieved in approximately 45–80% of IBD patients on maintenance anti-TNF therapy, and that this was not affected by alterations to vaccination scheduling around this therapy. PCV and PPV immunogenic responses have been shown to be impaired when on immunosuppressive therapy. PCV provides greater immune response than PPV and TNF inhibitors produce a greater inhibitory response than other immunosuppressive therapy [17]. This has provided weight to the approach to institute immunisation as soon as possible after diagnosis to ensure adequate response whilst not immunosuppressed [16, 17].

Data regarding response to other important vaccinations is less clear [24]. Antibody responses to tetanus and pertussis vaccination may be affected by immunosuppressive drug regimes, particularly the combination of immunomodulatory and anti-TNF agents [27], although studies are conflicting [24]. Owing to this uncertainty, recommendations have suggested vaccination with dTap before starting immunomodulators, particularly when they are used in combination with TNF inhibitor agents [27]. A dTap booster is routinely administered in secondary school (12–13 years) as part of the NIP, so optimising administration of this vaccine was important in our paediatric cohort.

There is a paucity of evidence to support specific strategies that improve vaccine efficacy in IBD patients. One adult trial comparing a standard HBV vaccination dose with a double dose regimen showed improved seroconversion in the double dose group [28]. Conversely, a randomised trial that compared one dose of seasonal influenza vaccine with a booster dose schedule in adult IBD patients being treated with immunomodulators and/or TNF inhibitor therapy found no improvement in influenza strain specific titres with a booster dose [29]. No studies have specifically addressed the question of optimal timing of vaccination administration in relation to IBD diagnosis.

The rate of travel to tuberculosis high-incident countries appeared high in our cohort at 24.6%. This is particularly important, as the reported frequency of tuberculosis activation in association with anti-TNF therapy is much higher than the reported frequency of other opportunistic infections associated with the drug [30]. Almost 60% of our cohort was screened for tuberculosis, with patients being treated with a TNF inhibitor more likely to undergo tuberculosis screening than other patients. Despite this, in both the travel to tuberculosis high-incident countries group (2/17, 11.8%) and the TNF inhibitor group (2/16, 12.5%) there were patients who did not receive tuberculosis screening.

A notable feature of this study was the high rate of additional vaccination uptake following SIC review. A Victorian audit previously conducted in 2007 showed low uptake of additional recommended vaccines in this group, with only 10% having ever had an influenza vaccination, and 5% an additional pneumococcal vaccination [14]. Comparatively, in this study 92.8% of patients received at least one vaccination following SIC review. Whilst influenza and pneumococcal vaccination prior to SIC was low at 24.6% and 8.7%, in the 12 months following SIC review these rates increased to 69.6% and 85.5% respectively. 43.5% of patients completed the additional recommended schedule within 12 months of SIC review. Interestingly, the vaccines that were most likely to be recommended but not received within the 12-month study follow-up period were PPV, HPV vaccine and dTap. Omission of PPV likely related to the AIH recommendation to delay this until at least 2 months after the PCV and subsequent loss of these patients within the follow-up system [13]. It is less clear as to why dTap and HPV vaccines were not administered and further investigation of this result is required. Nonetheless, these results emphasise the value of a SIC in assessing this special risk cohort.

Numerous barriers to immunisation in IBD patients have been reported, including cost. Several non-disease related reasons include access to up to date vaccination history and records, difficulties coordinating vaccination with primary care providers, poor access to vaccinations in the clinical setting, lack of awareness of routine vaccinations, needle phobia, and parental refusal [21, 31, 32]. A French study identified omission of proposal as the most common reason for delayed vaccination [31]. In a study assessing influenza vaccine uptake in IBD patients, fear of side effects and lack of belief in vaccine efficacy were the primary reasons affecting vaccine uptake [15]. Disease related reasons appear to primarily affect the administration of live vaccines, such as VZV, and include disease flare at the proposed time of vaccination and current use of immunosuppressive therapy [32]. Interestingly, a Canadian study found only 33% of incomplete immunisations in their IBD cohort to be due to disease related reasons [32].

One important outcome of this study was a change in our meningococcal vaccine recommendations. An increase in meningococcal serogroup W cases in Australia [33], highlighted a requirement to improve protection in adolescents with IBD, with 21.7% (15/69) having a meningococcal ACWY vaccine and 27.5% (19/69) a meningococcal B vaccine. The SIC therefore provided funding for these vaccines for patients on immunosuppressive therapy from April 2017 [34]. Additionally, in July 2017, the local Victorian Government funded a meningococcal ACWY vaccine for all 15–19 year olds [34]. These initiatives will continue to positively affect access to optimal immunisations in this special risk group into the future.

## Limitations

This study has several limitations. Firstly, the study did not fully assess anti-inflammatory and immunosuppressive therapies in detail, including dosing and duration, and timing of particular therapies in relation to timing of vaccinations. Secondly, we did not formally assess serological response to those vaccines administered during the study period. This information may have better guided our recommendations for further additional vaccines. Additionally, contraindications to certain vaccinations were not always documented. It is possible that some patients received their vaccinations outside of the 12-month follow-up window.

The presence of retrospective cases reduces the validity and accuracy of some of the data collected. Whilst recruitment of this data involved the utilisation of the RCH IBD database, the immunisation status of those diagnosed with IBD who have not been reviewed by the SIC was not assessed. This information would provide a useful comparison to further measure the impact of the SIC for this cohort.

## Conclusions

This study demonstrates that formal review by a SIC can help improve the uptake of additional recommended vaccines in a cohort of paediatric IBD patients. Development and enhancement of such services, including engagement with primary care providers, is necessary to better streamline immunisation assessment and provision. Additional research to improve our understanding of the mechanisms of serum immune responses to vaccination in this group would allow better optimisation of vaccine recommendations in the IBD cohort, with the aim of reducing the risk of vaccine preventable diseases.

## Data Availability

The datasets generated and analysed during this study are not publicly available due to the identifiable nature of the dataset, but are available from the corresponding author on reasonable request.

## Abbreviations

ACIR: Australian Children’s Immunisation Register
AEFI: Adverse events following immunisation
AIH: Australian Immunisation Handbook
AIR: Australian Immunisation Register
ASGS: Australian Statistical Geography Standard
BCG: Bacille Calmette–Guerin
dTap: Diphtheria, tetanus, acellular pertussis
HAV: Hepatitis A Virus
HBV: Hepatitis B Virus
HPV: Human Papilloma Virus
EMR: Electronic medical record
IBD: Inflammatory Bowel Disease
ImPS: Immunisation Program Service
IQR: Interquartile range
MCRI: Murdoch Children’s Research Institute
MMR: Measles Mumps Rubella
NIP: National Immunisation Program
NSAID: Non-steroidal anti-inflammatory drug
PCV: Pneumococcal conjugate vaccine
PPV: Pneumococcal polysaccharide vaccine
RedCAP: Research Electronic Data Capture
RCH: Royal Children’s Hospital
SAEFVIC: Surveillance of Adverse Events Following Vaccination in the Community
SIC: Specialist Immunisation Clinic
TB: Tuberculosis
TNF: Tumour necrosis factor
VZV: Varicella Zoster Virus
